# Biomarker-Associated Remission After Switching to Dupilumab in Severe Asthma Following Failure of Prior Biologics

**DOI:** 10.3390/biomedicines13092096

**Published:** 2025-08-28

**Authors:** Fabio Romano Selvi, David Longhino, Gabriele Lucca, Ilaria Baglivo, Maria Antonietta Zavarella, Chiara Laface, Laura Bruno, Arianna Delfino Spiga, Sara Gamberale, Ludovica Fabbroni, Angela Rizzi, Arianna Aruanno, Marina Curci, Alessandro Buonomo, Stefania Colantuono, Marinella Viola, Gianluca Ianiro, Antonio Gasbarrini, Cristiano Caruso

**Affiliations:** 1 UOSD Allergy and Clinical Immunology Unit, Fondazione Policlinico Universitario A. Gemelli, IRCCS, Università Cattolica del Sacro Cuore, 00168 Rome, Italyangela.rizzi@policlinicogemelli.it (A.R.);; 2 UOSD DH Internal Medicine and Digestive Disease, Fondazione Policlinico Universitario A. Gemelli, IRCCS, 00168 Rome, Italy; 3 Centre for Digestive Disease (CEMAD) and Gastroenterology Unit, Fondazione Policlinico Universitario A. Gemelli, IRCCS, 00118 Rome, Italy

**Keywords:** dupilumab, severe asthma, biologic switch, biomarkers, eosinophils, IgE, Eosinophilic Cationic Protein, type 2 inflammation

## Abstract

**Background/Objectives**: Severe asthma remains difficult to treat, even with the range of biologics we now have that target type 2 inflammation. Some patients do not respond well enough to the first biologic they try, which raises the question of whether switching to another option can help. In this study, we looked at how patients who had unsatisfactory therapeutic outcomes on other biologics responded—both clinically and at the biomarker level—after switching to dupilumab. **Methods**: We reviewed data from the Allergy and Clinical Immunology Unit of Fondazione Policlinico Universitario A. Gemelli-IRCCS, Rome, Italy, between January and June 2025. The study included fifteen adults with uncontrolled severe asthma who had previously been treated for at least six months with benralizumab, omalizumab, or mepolizumab before switching to dupilumab. We evaluated demographic, clinical and laboratory data. Lung function (Forced Expiratory Volume in 1 s (FEV_1_)), blood eosinophils, total and specific IgE to staphylococcal enterotoxins, eosinophil cationic protein (ECP), free light chains (FLC), and FeNO were assessed at the time of the switch and again after 12 months. Comparisons were made using paired tests, and a *p*-value < 0.05 was considered statistically significant. **Results**: After a year on dupilumab, we saw clear improvements: mean FEV_1_ went up by about 10.8% predicted (*p* = 0.002), FeNO dropped by an average of 22 ppb (*p* = 0.005), blood eosinophils fell by roughly 400 cells/µL (*p* = 0.003), and ECP levels decreased by 13 µg/L (*p* = 0.009). Kappa FLCs also showed a significant drop (*p* = 0.04). Clinically, 40% of patients met criteria for a meaningful response, and 20% achieved complete remission. Dependence on oral corticosteroids was notably reduced. Baseline levels of eosinophils, ECP, IgE, and FLCs correlated with response to treatment. **Conclusions**: Our study, despite the small sample size, highlights that in patients with severe asthma who do not show a good response to their first biologic, switching to dupilumab can lead to significant improvements. Markers of type 2 inflammation at baseline might help predict who benefits most. Larger, multi-center, prospective studies are needed to confirm these results.

## 1. Introduction

Severe asthma is defined by the ERS/ATS (European Respiratory Society/American Thoracic Society) 2014 [1] consensus as “asthma that requires treatment with high-dose inhaled corticosteroids (ICS) plus a second controller (and/or systemic corticosteroids) to prevent it from becoming uncontrolled, or which remains uncontrolled despite this therapy”. It continues to pose a significant and persistent therapeutic challenge for both patients and healthcare providers, despite the advent of a growing arsenal of targeted biologic therapies designed to address the underlying inflammatory mechanisms of the disease.

Although these biologics—each targeting different arms of type 2 inflammation—have expanded our treatment options considerably, approximately 3 to 5% of the total asthmatic population continues to meet criteria for severe, difficult-to-treat, or uncontrolled asthma [2].

This is despite adherence to guideline-recommended, high-intensity treatment regimens that include maximum doses of inhaled corticosteroids (ICSs) in combination with long-acting β_2_-agonists (LABAs), with or without long-acting muscarinic antagonists (LAMAs). Many of them continue to depend on daily oral corticosteroids (OCS), often leading to substantial long-term side effects [3] and a diminished quality of life.

In response to these challenges, the clinical practice of switching between biologics has become increasingly common in specialized asthma centers [4,5], particularly in cases where patients fail to achieve adequate control on a first-line agent like anti-IgE (omalizumab), anti-IL-5 (mepolizumab), or anti-IL-5R (benralizumab) therapies, and are therefore switched to anti-IL-4Rα agents such as dupilumab, which blocks both IL-4 and IL-13 signaling pathways.

This trend has been supported by data from large real-world observational studies, which report switch rates ranging from 15 to 20% in select populations [6]. Still, despite the growing frequency of these therapeutic shifts, hard data on the outcomes—both in terms of clinical improvement and changes in relevant biomarkers—remains relatively limited [7].

Among the existing biologics, omalizumab has long held a prominent place in the management of allergic asthma, with its ability to reduce exacerbation rates, improve pulmonary function, and enhance patients quality of life safely and effectively [8,9], it has secured its role as a well-established add-on therapy in patients who remain symptomatic despite optimized inhaled therapy [10]. Studies published on switching to dupilumab have demonstrated excellent efficacy in treating nasal and asthma symptoms, but research into new biomarkers is still limited when changing to a new biological treatment [11,12,13].

Current guidelines have emphasized the importance of a personalized, biomarker-driven strategy when initiating or modifying biologic treatment [14], considering factors such as blood eosinophil counts, serum IgE levels, FeNO, and specific allergen sensitivities.

Given the heterogeneity and complexity of type 2 inflammation in asthma, there is a solid clinical rationale for redirecting treatment toward a different immunologic pathway when one approach proves ineffective [15].

In light of all this, our study set out to evaluate the real-world impact of switching to dupilumab in a cohort of patients with severe asthma who had experienced suboptimal outcomes with a prior biologic. Specifically, we aimed to assess not only symptomatic and functional improvements but also changes in key biomarkers, using retrospective data collected from our Allergology and Clinical Immunology unit.

## 2. Materials and Methods

### 2.1. Study Design and Participants

This was a retrospective, observational study carried out at the Allergy and Clinical Immunology Unit of Fondazione Policlinico Universitario A. Gemelli-IRCCS between January and June 2025. We used the ERS/ATS international guidelines to define severe asthma [1], and followed the STROBE recommendations for reporting observational data [16].

Fifteen adults (12 women, 3 men) with uncontrolled severe asthma were included after signing informed consents. All had been on at least 6 months of a previous biologic—benralizumab (*n* = 7), omalizumab (*n* = 4), or mepolizumab (*n* = 4)—without adequate symptom control. The decision to switch to dupilumab was made as part of routine clinical care, based on ongoing symptoms and shared decision-making with patients.

Given the small sample size, parity in the number of patients between the two sexes has not been reached.

### 2.2. Data Collection and Outcomes

We reviewed clinical and laboratory data at the time of switch (t_0_) and again 12 months later (t_12_). We looked at the following variables:

- Forced expiratory volume in one second (FEV_1_ % predicted)
- Blood eosinophil counts, total IgE, and eosinophil cationic protein (ECP)
- Serum free light chains (kappa and lambda)
- Total and specific IgE to staphylococcal enterotoxins
- Fractional exhaled nitric oxide (FeNO)

All baseline samples were collected at least 8 weeks after the last dose of the previous biologic to avoid overlap effects.

For each patient, we used the therapeutic doses of dupilumab normally administered in clinical practice for asthma (since all patients were adults, an initial dose of 600 mg, followed by a dose of 300 mg every two weeks).

### 2.3. Statistical Analysis

We reported continuous data as mean ± standard deviation or median with interquartile range, and categorical data as counts and percentages. The Shapiro–Wilk test was used to check for normal distribution. Paired *t*-tests or Wilcoxon signed-rank tests were used to compare data between t_0_ and t_12_. Correlations were assessed using Pearson or Spearman coefficients, depending on distribution. A *p*-value < 0.05 was considered statistically significant. Analyses were performed with SPSS v25.0. Agreement statistics followed the Bland & Altman method [17].

## 3. Results

**Figure 1:**
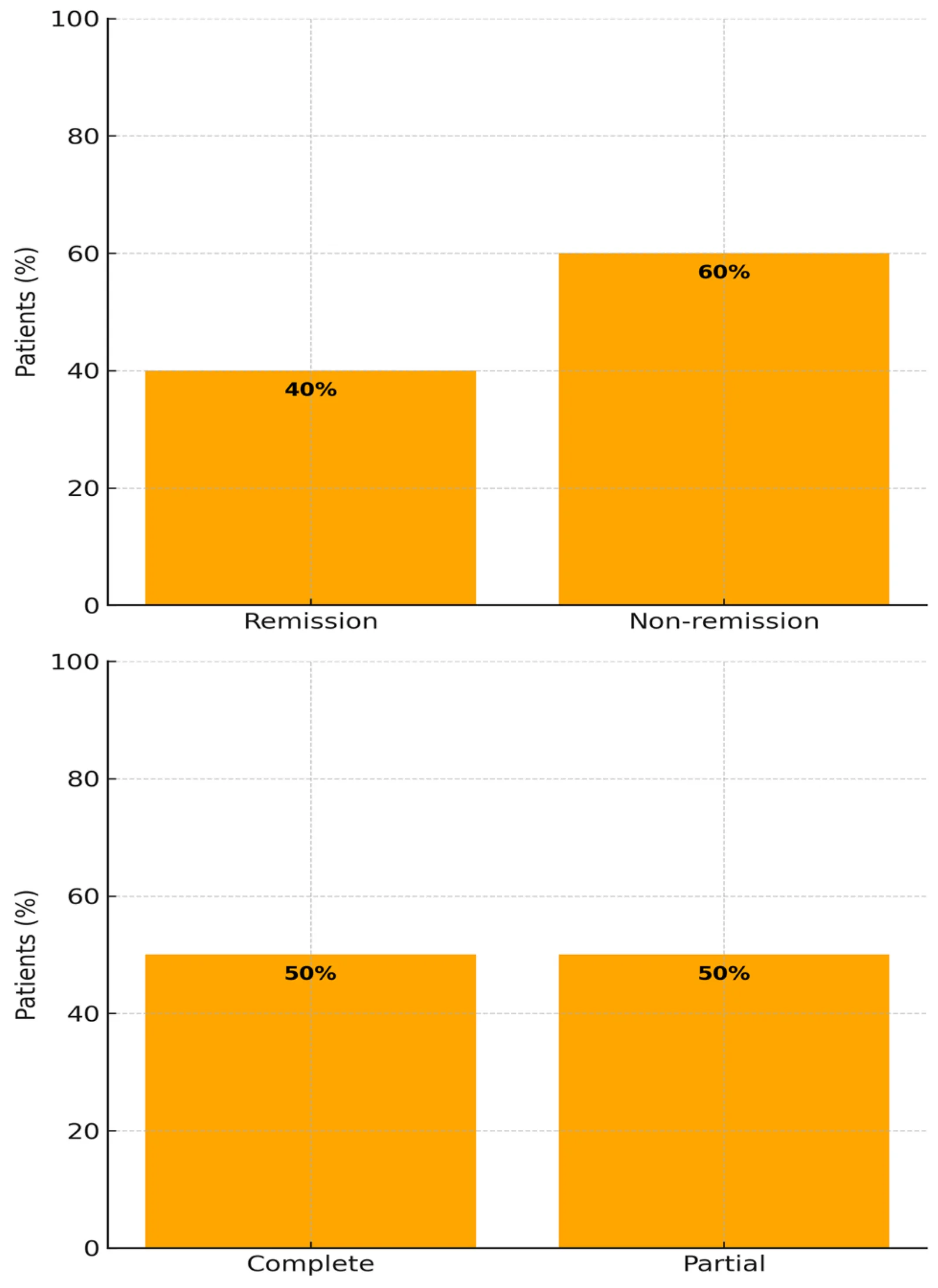
Distribution of remission after switching to dupilumab, according to the SANI definition. The upper panel shows the proportion of patients who achieved remission (complete or partial) versus those who did not. The lower panel specifies the distribution between complete remission (*n* = X) and partial remission (*n* = Y). *Y*-axis values are expressed as percentages (%).

A clinically meaningful response—defined as either a ≥10% gain in FEV_1_ or a ≥50% drop in eosinophils—was seen in 6 out of 15 patients (40%), with 3 achieving partial remission, and 3 patients reaching complete remission (according to the SANI definition), as shown in the following image (Figure 1).

After 12 months of treatment with dupilumab, we observed significant improvements across several key clinical and inflammatory markers. These changes are summarized in Table 1 and discussed below:

- Forced expiratory volume in one second (FEV_1_) increased by a mean of 10.8% predicted (*p* = 0.002), indicating a substantial improvement in lung function.
- Fractional exhaled nitric oxide (FeNO) levels dropped by an average of 22 parts per billion (ppb) (*p* = 0.005), suggesting a reduction in airway inflammation.
- Peripheral blood eosinophil counts decreased by approximately 400 cells/µL (*p* = 0.003), consistent with reduced systemic eosinophilic inflammation.
- Eosinophil cationic protein (ECP) levels fell by 13 µg/L (*p* = 0.009), further supporting the anti-inflammatory effects of dupilumab [18].
- Kappa free light chains (FLCs), which have been proposed as novel biomarkers in severe asthma, also showed a significant mean reduction of 2.5 mg/L (*p* = 0.04) [19].

Taken together, these data reflect a clear biomarker and functional response to dupilumab in this difficult-to-treat cohort. In terms of clinical outcomes, a meaningful response, defined as either a ≥10% gain in FEV_1_ or a ≥50% reduction in blood eosinophils, was observed in 6 out of 15 patients (40%). Among these responders, 3 individuals achieved partial remission, while the remaining 3 met the criteria for complete remission, based on the SANI (Severe Asthma Network Italy) definition. These rates are visually summarized in Figure 1 and are noteworthy, given the previous lack of efficacy with other biologics.

Additionally, we performed correlation analyses to explore the relationships among baseline biomarkers. Blood eosinophil counts were strongly correlated with both ECP (rho = 0.84) and kappa FLCs (rho = 0.81), with *p* < 0.001 for both associations. Similarly, total IgE levels exhibited a robust correlation with kappa FLCs (rho = 0.80, *p* < 0.001). These strong correlations reinforce the potential utility of these biomarkers in predicting and monitoring response to treatment.

**Figure 2:**
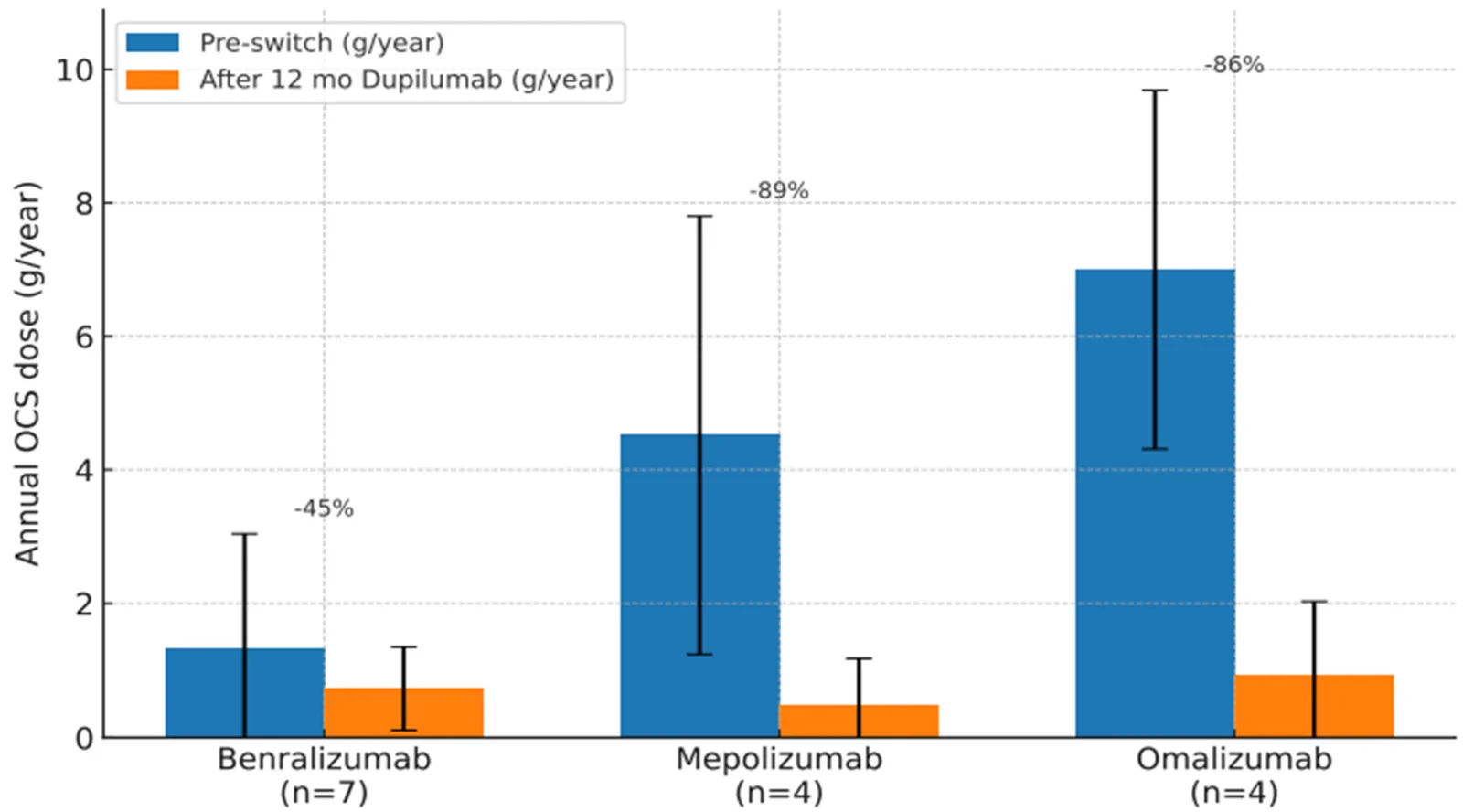
Effect of dupilumab on oral corticosteroid (OCS) use. Annual OCS dose (g/year) is shown before switching (blue) and after 12 months of dupilumab therapy (orange). *Y*-axis represents OCS dose in grams per year (g/year). Substantial reductions were observed across all groups, particularly among patients previously treated with mepolizumab and omalizumab.

Oral corticosteroid (OCS) use decreased substantially after the switch. Mean annual OCS dose fell from 1.33 ± 1.71 to 0.73 ± 0.62 g/year in the benralizumab group (≈45% reduction), 4.52 ± 3.28 to 0.48 ± 0.70 g/year in the mepolizumab group (≈89%), and 7.00 ± 2.69 to 0.95 ± 1.08 g/year in the omalizumab group (≈86%). These declines were accompanied by fewer exacerbations (e.g., mean annual exacerbations decreased from 4.5 to 0.5 in the benralizumab group, 3.6 to 0.0 in mepolizumab, and 4.25 to 1.0 in omalizumab) and improved control (higher ACT and FEV_1_), creating conditions for tapering. In our real-life setting, clinicians pursued steroid-sparing whenever patients maintained good control, reducing maintenance OCS stepwise. The larger absolute OCS reductions in the omalizumab and mepolizumab groups primarily reflect higher baseline OCS exposure, whereas relative reductions were observed across all groups. Figure 2 summarizes the changes in annual OCS dose by prior biologic class and the magnitude of reduction after 12 months of dupilumab.

## 4. Discussion

Our findings align closely with those reported in major clinical trials investigating dupilumab for the treatment of type 2 severe asthma, which have consistently demonstrated significant improvements in lung function (as measured by FEV_1_), alongside a marked reduction in oral corticosteroids dependency [20,21]. A growing body of real-world evidence also supports the effectiveness of dupilumab in patients who have previously received other biologic therapies, such as anti-IgE or anti-IL-5/5R agents [13,22,23,24]. These studies reflect the complexities of everyday clinical practice and reinforce the notion that therapeutic switching—particularly to a drug with a different mechanism of action—can be a valuable strategy in cases of suboptimal response. In our own clinical experience, the process of switching to dupilumab was conducted safely and effectively without the implementation of a washout period between biologics, as no serious adverse events were observed during the transition [12]. However, we did observe a few cases of transient eosinophilia in the early phases of treatment, which underscores the importance of ongoing clinical and laboratory monitoring during the switch, especially in patients with high baseline eosinophil counts [25]. Serum interleukin levels were not monitored, as ours is a clinically oriented study and the measurement of serum levels is not currently a routine test. Our data add to the growing recognition that specific biomarkers, such as blood eosinophil levels, fractional exhaled nitric oxide (FeNO), serum IgE, and free light chains (FLCs), are likely valuable predictors for identifying patients who are more likely to respond favorably to dupilumab [26]. Therefore, including these biomarkers into routine assessment may enable for a more tailored approach to biologic selection and switch effectiveness. Some comparative studies also suggest that biologic treatment with dupilumab might result in fewer exacerbations and lower steroid requirements when compared to other biologic agents targeting IL-5 or IgE [27,28,29]. It should, however, be noted that previous studies have reported similar improvements when switching from anti-IgE therapies like omalizumab to anti-IL-5 agents such as mepolizumab or benralizumab, further validating this strategy across different biologic classes [30,31]. Overall, these findings support a nuanced, stepwise approach to asthma management—one that is guided by biomarkers, tailored to each patient’s inflammatory profile, and thus can yield meaningful clinical improvements even after prior biologic failure.

## 5. Limitations

This was a small, single-center, retrospective study, so the findings should be interpreted with caution. Larger prospective trials that stratify patients based on biomarkers will be key to confirming these results [32,33].

## 6. Conclusions

In this real-world cohort of patients with severe asthma, transitioning to dupilumab after the failure of a prior biologic therapy resulted in clinically meaningful improvements, both in terms of symptom control and biomarker modulation. These findings suggest that a switch to dupilumab can be a valuable therapeutic option in cases of inadequate response to other biologics. Moreover, elevated baseline levels of type 2 inflammatory markers, particularly blood eosinophils, eosinophil cationic protein (ECP), total IgE, and serum free light chains (FLCs), appear to be associated with a more favorable response, highlighting their potential role as predictive tools in guiding individualized treatment strategies.

## Figures and Tables

**Table 1:** Clinical and biomarker results before and after 12 months of dupilumab therapy. Data are expressed as mean ± standard deviation. Abbreviations: Benra = benralizumab, Mepo = mepolizumab, Oma = omalizumab, Dupi = dupilumab. “Post Benra/Before Dupi,” “Post Mepo/Before Dupi,” and “Post Oma/Before Dupi” indicate results obtained after treatment with the previous biologic but before switching to dupilumab. “After 1y Dupi (Benra/Mepo/Oma)” indicates results after 12 months of dupilumab therapy in patients previously treated with that biologic.

	T0	Post Benra/ Before Dupi	Post Mepo/ Before Dupi	Post Oma/ Before Dupi	After 1y Dupi (Benra)	After 1y Dupi (Mepo)	After 1y Dupi (Oma)
Male, *n* (%)	3 (20%)						
Female, *n* (%)	12 (80%)						
Age, mean ± SD	59.87 ± 15.84						
Sensitization to m223, *n* (%)	2 (13.3%)						
Sensitization to m226	6 (40%)						
Sensitization to m81, *n* (%)	5 (33.3%)						
FEV1 (%), mean ± SD	56.8 ± 10.32	62 ± 5.89	62.6 ± 9.77	61.75 ± 7.94	83.5 ± 4.89	80.2 ± 7.22	78 ± 9.02
FeNO (ppb), mean ± SD	63 ± 17.78	56.67 ± 10.18	51.2 ± 17.07	78.25 ± 19.90	27.83 ± 9.24	32.6 ± 18.49	63 ± 11.42
Eosinophils (cells/mcl), mean ± SD	191.6 ± 268.77	16.33 ± 12.62	59.2 ± 5.56	620 ± 138.16	156.67 ± 60.98	226 ± 43.63	342.5 ± 66.84
Total IgE (kU/L), mean ± SD	382.33 ± 404.26	182 ± 130.68	208.6 ± 152.69	900 ± 438.4	90.17 ± 57.57	101.6 ± 61.78	443.75 ± 63.97
Kappa Free-Light-Chains (mg/L)	23.87 ± 6.21	19.8 ± 4.06	22.4 ± 3.92	31.82 ± 3.18	11.77 ± 2.18	13.36 ± 2.32	17.75 ± 2.51
Lambda Free-Light-Chains (mg/L)	25.79 ± 7.61	22.5 ± 4.74	25.26 ± 4.40	31.37 ± 10.54	13.22 ± 2.80	14.14 ± 2.46	17.47 ± 6.38
ECP (µg/L)	31 ± 20.36	13.58 ± 5.06	28.12 ± 4.06	60.9 ± 11.69	20.38 ± 6.20	24.20 ± 5.64	38.25 ± 7.49
Exacerbations	4.13 ± 2.31	4.5 ± 2.5	3.6 ± 0.8	4.25 ± 3.03	0.5 ± 0.5	0.0 ± 0.0	1 ± 1.22
OCS dose (g/y)	3.91 ± 3.47	1.33 ± 1.71	4.52 ± 3.28	7 ± 2.69	0.73 ± 0.62	0.48 ± 0.70	0.95 ± 1.08
ACT, mean ± SD	13.6 ± 4.9	13 ± 3.56	17.12 ± 4.06	10.75 ± 4.14	22.6 ± 2.33	20.6 ± 3.61	21.25 ± 1.78

## Data Availability

The original contributions presented in this study are included in the article. Further inquiries can be directed to the corresponding author.
